# Recent Advances in Preclinical Studies of the Theranostic Agent [^64^Cu]CuCl_2_

**DOI:** 10.3390/molecules29174085

**Published:** 2024-08-28

**Authors:** Giorgia Speltri, Francesca Porto, Alessandra Boschi, Licia Uccelli, Petra Martini

**Affiliations:** 1 Department of Chemical, Pharmaceutical and Agricultural Sciences (DoCPAS), University of Ferrara, 44121 Ferrara, Italy; giorgia.speltri@unife.it; 2 Department of Translational Medicine, University of Ferrara, 44121 Ferrara, Italy; francesca.porto@unife.it (F.P.); licia.uccelli@unife.it (L.U.); 3 Department of Environmental and Prevention Sciences, University of Ferrara, 44121 Ferrara, Italy; petra.martini@unife.it

**Keywords:** theranostic, Cu^2+^ ion, preclinical studies

## Abstract

^64^Cu is gaining recognition not only for its diagnostic capabilities in nuclear medical imaging but also for its therapeutic and theranostic potential. The simultaneous βˉ and Auger emissions of ^64^Cu can be utilized to induce a therapeutic effect on cancerous lesions. The finding of the exceptional biodistribution characteristics of the radionuclide ^64^Cu, when administered as basic copper ions, has highlighted its potential therapeutic application in cancer treatment. Preclinical and clinical research on the effectiveness of [^64^Cu]CuCl_2_ as a theranostic radiopharmaceutical has commenced only in the past decade. Current clinical studies are increasingly demonstrating the high specificity and uptake of [^64^Cu]Cu^2+^ by malignant tissues during early cancer progression, indicating its potential for early cancer diagnosis across various organs. This short review aims to present the latest preclinical studies involving [^64^Cu]CuCl_2_, offering valuable insights for researchers planning new in vitro and in vivo studies to explore the theranostic potential of [^64^Cu]Cu^2+^.

## 1. Introduction

**Figure 1:**
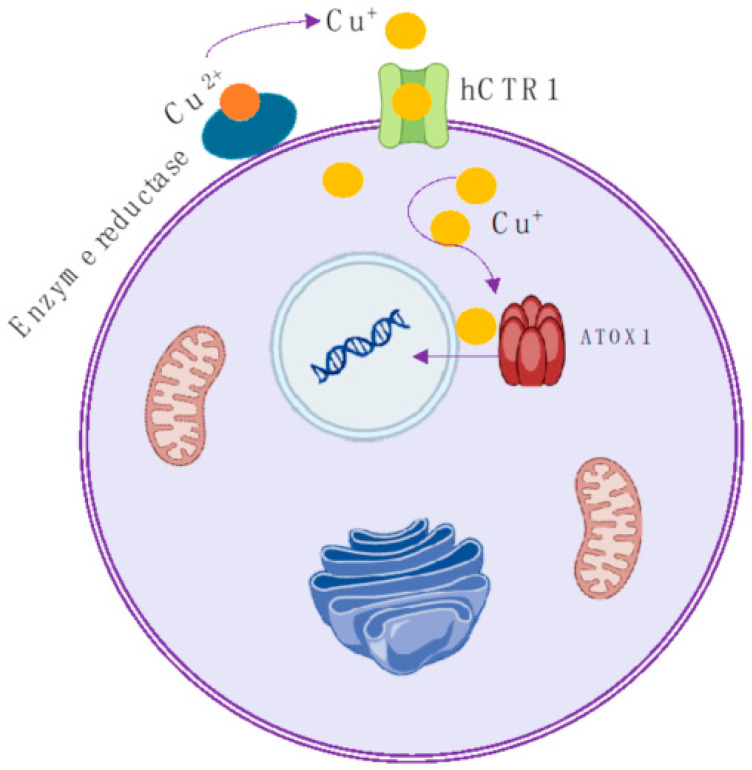
Schematic representation of the copper cellular uptake. Cu^2+^ is reduced to Cu^+^ outside the cell membrane by reductase enzymes, then enters inside the cells using the enzyme CTR1. Subsequently, it penetrates the nucleus and integrates into the DNA with the aid of the enzyme ATOX1. There is a distinct difference in behavior between normal and tumor cells. In normal cells, ^64^Cu stays within the cytoplasm, whereas in tumor cells, it makes its way into the nucleus [3].

Copper is a transition metal essential for the function of numerous molecules that regulate various metabolic pathways in human physiology [1]. In recent decades, significant progress has been made in understanding the molecular and cellular mechanisms that regulate copper balance. More recently, copper has been identified as playing a pivotal role in maintaining healthy neuronal function. Concurrently, abnormal copper regulation has been observed in numerous significant and prevalent diseases such as Parkinson’s disease, Alzheimer’s disease, motor neuron diseases, amyotrophic lateral sclerosis, and prion diseases. Moreover, it plays a role in signaling transduction pathways that regulate cancer cell proliferation and tumor growth [2]. In particular, recently, it has been shown that the radionuclide ^64^Cu, in its basic form as Cu^2+^ ion, is specifically absorbed by various cancerous tissues and eventually integrated into the cell nucleus in close proximity to DNA. The key stages of the copper ion uptake process into the cell nucleus have also been clarified [1,3]. Essentially, in the bloodstream, Cu^2+^ ions bind to plasma proteins (ceruloplasmin, albumin, and transcuprein), which transport them to the cell surface where enzymes (reductases) reduce Cu^2+^ ions to Cu^+^ ions before they are absorbed into cells (Figure 1). In their reduced state, Cu^+^ ions are then transported across the cell membrane by the copper transporter 1 (CTR1) and enter the cell, where they are tightly bound by various copper chaperone proteins (COX17, CCS, and antioxidant protein ATOX1) that deliver Cu^+^ ions to the cytosol, mitochondria, and the trans-Golgi network. Within the cell, copper plays a fundamental role in regulating cell growth by influencing gene expression. As recently observed, this occurs when the activated protein ATOX1 also acts as a transporter of copper ions to the nucleus, where it contributes to cell proliferation [4]. This behavior explains the observation that highly proliferating cancerous tissues have higher levels of copper than normal tissues. Based on this mechanism, it is evident that selecting Cu^2+^ ions as chemical carriers for copper radioisotopes may enable the targeting of subcellular structures.

Recent advancements in positron emission tomography and X-ray computed tomography (PET/CT) now enable non-invasive, real-time analysis of copper metabolism in extrahepatic tissues. This allows for the evaluation of various pathological conditions and diseases affecting copper metabolism, including hereditary defects such as Menkes syndrome and Wilson’s disease, as well as acquired copper metabolism disorders or imbalances caused by factors like pregnancy, inflammation, tumor growth, and metastasis [5].

^64^Cu is emerging not only for its diagnostic potential in nuclear medical imaging but also for its therapeutic and thus theranostic potential. The concomitant βˉ and Auger emissions of ^64^Cu can be exploited to trigger a therapeutic effect on cancerous lesions [6]. The administration of ionic [^64^Cu]Cu^2+^ in a physiological solution allows for the selective targeting of a variety of neoplasms, due to the natural role of copper ions in cellular proliferation, without being involved in inflammatory processes [7].

The discovery of the remarkable biodistribution properties of the radionuclide ^64^Cu, administered as simple copper ions, has also revealed its possible therapeutic use for cancer treatment. However, experiments conducted with [^64^Cu]CuCl_2_ have so far demonstrated that this therapeutic potential can be fully exploited only when the radionuclide accumulates within the cell nucleus in close proximity to the genetic material. Indeed, Auger electrons emitted in the immediate vicinity of the DNA double helix could produce the highest therapeutic effect through the irreversible breakage of the genetic material of cancer cells, causing biological damage similar to that caused by heavier particles with a higher LET, such as alpha particles.

Leveraging the key role of copper ions in the replication and uncontrolled growth of cancer cells, the administration of radioactive ionic copper [^64^Cu]CuCl_2_ allows it to exert its therapeutic function after nuclear internalization by cancer cells.

Preclinical and clinical investigations to evaluate the effectiveness of [^64^Cu]CuCl_2_ as a theranostic radiopharmaceutical have only begun in the last 10 years. Ongoing clinical studies are progressively revealing the high specificity and uptake of [^64^Cu]Cu^2+^ by malignant tissues in the early stages of cancer progression, highlighting its potential for early diagnosis of cancerous lesions in various organs. 

The aim of this short review is to provide state-of-the-art of preclinical studies conducted with [^64^Cu]CuCl_2_, useful for researchers intending to carry out new in vitro and in vivo preclinical studies to highlight the theranostic potential of the [^64^Cu]Cu^2+^ ion. The following sections will summarize the [^64^Cu]CuCl_2_ radiochemistry and the preclinical studies conducted so far in the diagnosis and treatment of different cell lines and animal models of various pathologies with [^64^Cu]CuCl_2_.

## 2. Copper Radiochemistry

### 2.1. ^64^Cu Production

Among copper radioisotopes (Table 1), ^64^Cu and ^67^Cu have for years been in the spotlight of the scientific community as excellent candidates for therapeutic applications. Both diagnosis and therapy can be performed using the same radiolabeled species using the combination of therapeutic (β- Auger particles and electrons) and diagnostic (β^+^ or γ) emissions.

The main limiting factor for a wider application of ^67^Cu in medical practices, at present, is the fairly modest overall availability, while ^64^Cu availability and cost of production have attracted the interest of the scientific community [8].

^64^Cu is produced using low to medium-energy medical cyclotrons (16–19 MeV) through the following nuclear reaction: ^64^Ni(p,n)^64^Cu [9]. Other production methods using cyclotrons involve reactions induced by protons or deuterons, such as ^64^Ni(d,2n)^64^Cu, ^68^Zn(p,αn)^64^Cu, ^66^Zn(p,2pn)^64^Cu, ^64^Zn(d,2p)^64^Cu, ^66^Zn(d,α)^64^Cu, and ^67^Zn(p,α)^64^Cu, or by neutrons in a nuclear reactor, such as ^64^Zn(n,p)^64^Cu [6,10].

In the production of ^64^Cu from the nuclear reaction ^64^Ni(p,n)^64^Cu, the target can be (i) solid, using ^64^Ni enriched and electroplated on a gold coin (called backing); (ii) liquid, a solution of salts of ^64^Ni enriched in nitric acid [11].

A critical factor in the production of copper isotopes for medical purposes is to minimize the dilution of the isotope of interest with non-radioactive copper from external sources. It is difficult to achieve high molar activity as copper is a ubiquitous element [12]. To prevent metal contamination at all stages of target preparation and handling, it is recommended that instruments and accessories be used exclusively for the production of ^64^Cu in order to avoid cross-contamination by other isotopes produced in addition to the use of metal-free solvents [13]. For the irradiation of the liquid target, it is recommended to use irradiation stations covered by a coating of niobium that, due to its inertia, has been proven to be a suitable material to house the acid solution of nickel in for the production of ^64^Cu.

### 2.2. ^64^Cu Separation and Purification

Following irradiation of the target, the methods used to separate the ^64^Cu produced from the target and other contaminants are the same in the case of solid and liquid targets, with the only difference being that for solid targets, a solid nickel dissolution phase is required. Dissolution of the irradiated solid target involves a heat treatment at 90 °C with a solution of HCl 9M. The resulting solution is allowed to evaporate, increasing the temperature to 95 °C, and the residue resumed with fresh HCl 9M. The solution containing ^64^Ni, ^64^Cu, and other contaminants is ready for the radiochemical separation process. Separation and purification of ^64^Cu is typically conducted with ion exchange resins (in anionic or cationic form, e.g., AG1-X8, or AG50W-X8, Bio-Rad, Hertfordshire, UK) for differential elution of Ni, Co, and Cu at different acid concentrations (typically HCl or HNO_3_). This process is simple and easy to automate [14].

Jauregui–Osoro et al. in 2021 [14] described an example of a radiochemical process for the separation and purification of the cyclotron produced ^64^Cu, by a solid nickel target, based on the use of an anionic exchange resin. The target, previously dissolved in HCl 9M was directly loaded onto a chromatographic column (0.7 × 20 cm) filled with 3.5 g of AG1X8 resin (chloride form, 100–200 mesh) preconditioned with HCl 6M. After washing the column with HCl 6M to elute excess ^64^Ni and HCl 4M to remove any impurities of radiocobalt, ^64^Cu was eluted with HCl 0,1M.

The separation mechanism in ion exchange chromatography is based on the formation of the CuCl_3_^−^ and CuCl_4_^2−^ anionic chlorocomplexes in hydrochloric acid, as illustrated in the equations below (which neglect the presence of coordinated water molecules):

Cu^2+^ + Cl^−^ ↔ CuCl^+^

CuCl^+^ + Cl^−^ ↔ CuCl_2_

CuCl_2_ + Cl^−^ ↔ CuCl_3_^−^

CuCl_3_^−^ + Cl^−^ ↔ CuCl_4_^2−^

The formation constant for each passage, the preferred coordination number of the metal, and the concentration of chloride ions determine the formation of cationic, neutral, or anionic species. the CuCl_3_^−^ and CuCl_4_^2−^ complexes predominate in HCl 9M, and bind to the anionic exchange column, allowing ^64^Cu to be purified from ^64^Ni and other cold and/or radioactive metal contaminants [14]. CuCl_2_ and CuCl^+^ do not interact with resin. As the concentration of HCl decreases, the equilibria shown above shift to the left, changing the net charges of the different metal chloro-complexes from negative to neutral or positive, thereby releasing the complexes from the resin sequentially [14].

Nickel does not form negatively charged complexes in appreciable amounts, even in highly concentrated hydrochloric acid; for this reason, nickel does not interact with anion exchangers [15].

After the separation and purification of the ^64^Cu from the target, the Ni eluted from the resin can be recovered with over 90% yield through a few evaporation and dissolution steps. This process allows the re-establishment of the electroplating conditions, which may vary from nitrate or nickel sulfate at pH 9 to nickel chloride at pH 7. To obtain the high molar activity radionuclide, a pre-purification of the target material before electroplating is required. This pre-purification should be conducted on both new enriched material and the recovered material using a CU resin [16].

**Figure 2:**
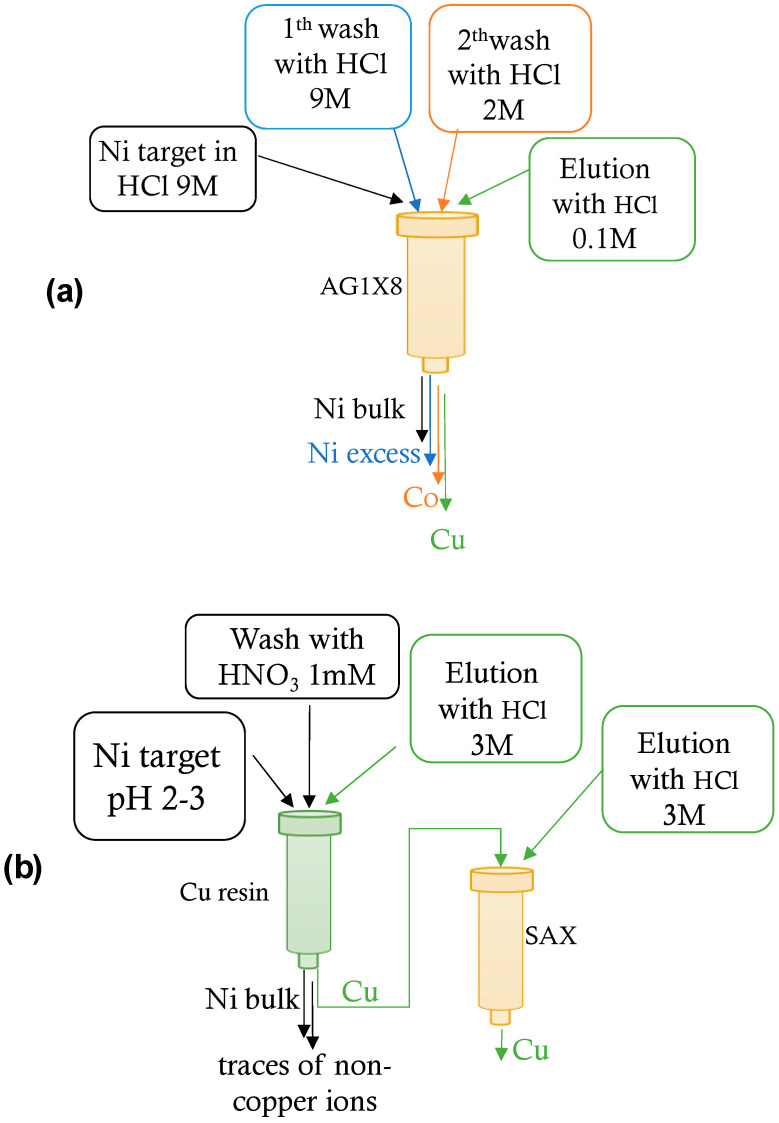
(**a**) Schematic representation of the separation and purification of the cyclotron produced ^64^Cu, by a solid nickel target (**a**) and by a liquid nickel target (**b**) [14].

In the case of a liquid target, a two-step automatic separation and purification procedure has been proposed. This involves the use of a highly selective Cu-resin (TrisKem International, Bruz, France) followed by a strong anion exchange resin [17]. The proposed separation procedures are schematized in Figure 2.

## 3. Preclinical Studies

One of the primary challenges in developing ^64^Cu-radiopharmaceuticals is ensuring their stability in vivo to prevent copper from binding with the numerous copper-chelating proteins in the blood. This has led to significant research into developing effective chelating agents for these radiopharmaceuticals. Among the chelating systems studied for the formation of stable copper complexes, sarcophagine (Sar) and its derivatives have so far proven to be the most suitable [10]. Hicks et al. [18] developed a new series of Sar-based somatostatin type 1 agonist bifunctional ligands derived from octreotide (SarTaTe). The preclinical evaluation of ^64^Cu-labeled SarTaTe demonstrated remarkable in vitro kinetic and thermodynamic stability of the ^64^Cu-conjugates, along with similar stability in biological fluids and favorable pharmacokinetic properties [18].

CTR1, a protein overexpressed in various cancer cells, plays a crucial role in the accumulation of [^64^Cu]CuCl_2_ in tumor cells, as evidenced by numerous studies reported in the following section across different cancer types, including prostate, breast, melanoma, glioblastoma, and lung cancer. This suggests a potential new era for theranostic applications of ^64^Cu, particularly in the simple form of copper chloride. Compared to ^64^Cu-labeled conjugates, [^64^Cu]CuCl_2_ offers several benefits: easy production using compact biomedical cyclotrons, no need for complex radiolabeling processes, and clinical versatility with high stability and bioactivity toward CTR1. 

Future clinical applications of [^64^Cu]CuCl_2_, particularly in therapeutic procedures, require precise dose estimates, for which biodistribution data are necessary.

Manrique–Arias et al. [19] reported the biodistribution of [^64^Cu]CuCl_2_ in healthy rats and the estimation of radiation doses to humans by extrapolating the animal data. Micro-PET imaging demonstrated a rapid clearance of the radionuclide from the blood and a predominant uptake in the kidneys, liver, and bowels. This finding aligned with the data obtained from the biodistribution assays. The highest uptake was observed in the kidneys, with a value of (3.58 ± 1.18) %ID/g at 30 min post-injection, which gradually decreased to (0.88 ± 0.23) at 24 h. In the liver, uptake peaked at (1.92 ± 0.81) %ID/g at 3 h post-injection, then declined to (0.33 ± 0.07) at 24 h. The dosimetry results suggest that the lower large intestine wall is the critical organ, with absorbed doses reaching 139 ± 34 mGy/MBq for females and 125 ± 32 mGy/MBq for males. The estimated effective doses were 47 ± 4 mSv/MBq for females and 39 ± 4 mSv/MBq for males.

However, it must be considered that studies have shown that the biodistribution of copper in tumor-bearing animals differs from that in corresponding control animals [20] and this may also apply to humans. Therefore, dose estimates based on biodistribution data from cancer patients might differ from those obtained from healthy individuals.

It is still unclear whether ^64^Cu in the form of [^64^Cu]CuCl_2_ will be utilized as a theranostic agent in humans. However, the inconsistencies in the current dosimetric data for this tracer highlight the need for a dosimetric study involving healthy volunteers to accurately determine safe dose levels and identify the organs most susceptible to dose limitations. This is especially crucial for therapeutic uses, as the primary goal of targeted radionuclide therapy is to deliver a sufficient dose to eliminate tumors while minimizing damage to other essential organs.

### 3.1. Prostate Cancer

^64^Cu, in its simplest form of [^64^Cu]CuCl_2_, which does not need complexation with targeting ligands, has recently been proposed as a promising agent for prostate cancer therapy, based on preclinical studies on cellular and animal models, as well as the increasing number of human studies documenting its use for prostate cancer diagnosis [21]. [^64^Cu]CuCl_2_ exhibited a swift uptake in the liver and renal cortex 10 min after administration. It is neither excreted through the urinary tract nor accumulated in the bladder, this results in a favorable biodistribution for detecting prostate tumors [22]. Patients showed no ureter and bladder uptake of [^64^Cu]CuCl_2_; thus, abdominal or pelvic lesions could be easily visually detected. No adverse or clinically noticeable pharmacologic effects were observed [21].

The study conducted by Peng et al. [23] demonstrated that prostate cancer that recurs locally may be detected using [^64^Cu]CuCl_2_ as a tracer. They used human prostate cancer cells (PC-3, ATCC) grown in RPMI 1640 medium with 10% fetal bovine serum, penicillin (100 U/mL), and streptomycin (100 mg/mL), and male athymic mice (4–5 weeks old, weighing 21.5–25.0 g). PC-3 cells (5 million per injection site) were injected subcutaneously into the right flank of the mice. The mice with tumors were then subjected to PET scans once the tumor xenografts reached approximately 0.8 × 0.8 cm in size. Athymic mice with human prostate cancer xenografts were imaged using PET at 1 h or 24 h following intravenous administration of [^64^Cu]CuCl_2_. PET imaging revealed elevated levels of hCTR1 after 24 h, but not after 1 h. They found a high level of tracer activity in the liver (17.5 ± 3.9%ID/g), with minimal tracer activity noted in the urinary bladder area. PET quantitative analysis revealed an increased uptake of [^64^Cu]CuCl_2_ in the tumor (3.6 ± 1.3%ID/g) 24 h post-injection. This uptake was approximately six times higher than that observed in the normal region (0.6 ± 0.3%ID/g), confirming that tumor tissues had a higher concentration of [^64^Cu]CuCl_2_ compared to normal soft tissues. The fact that human prostate xenografts were visible on images taken 24 h after injection but not on those taken 1 h after injection contrasts with the behavior observed in previously reported mouse hepatoma xenografts [24]. This discrepancy may be attributed to lower hCtr1 expression in prostate cancer compared to hepatoma, and the extended time required for tracer accumulation, which is influenced by a balance between tracer uptake via hCtr1 and washout via copper efflux pumps such as ATP7A and ATP7B. In a diagnostic clinical context, patients might undergo [^18^F]FDG PET imaging for metastasis detection on the first day, followed by injection with [^64^Cu]CuCl_2_ for emission scanning the next day to localize tumor lesions in the prostate area.

In a subsequent study by the same researchers, RNA interference was used to knock down hCtr1 to assess whether [^64^Cu]CuCl_2_ uptake is mediated by hCtr1 or if ionic copper binds nonspecifically to tumor tissue. For this purpose, a lentiviral vector containing short-hairpin RNA targeting hCtr1 (Lenti-hCtr1-shRNA) was designed to achieve RNA interference-mediated reduction of hCtr1 expression in prostate cancer cells. The extent of hCtr1 knockdown was evaluated using Western blot analysis, and its impact on copper uptake and cell proliferation was investigated in vitro through ^64^Cu uptake assays and cell proliferation tests. The impact of hCtr1 knockdown on tumor copper uptake was assessed using PET quantification and tissue radioactivity measurements. After 24 h, the uptake of [^64^Cu]CuCl_2_ by tumor cells lacking hCtr1 expression was significantly lower than in control tumor cells that expressed hCtr1 [25].

In a clinical scenario, ^64^Cu PET could therefore provide additional information for the clinical management of prostate cancer: The expression level of hCtr1 may correlate with the aggressiveness or prognosis of prostate cancer, and hCtr1 expression could be linked to the response of prostate cancer to cisplatin chemotherapy, as hCtr1 has recently been reported to mediate cellular uptake of cisplatin [26].

Guerreiro et al. (2018) [27] examined a variety of prostate cancer (PCa) cell lines derived from different metastatic sites—bone (22RV1, PC3, and VCaP), brain (DU145), and lymph node (LNCaP)—and used an immortalized, non-cancerous prostate cell line (RWPE-1) as a control. They integrated cytogenetic techniques with radiocytotoxicity assays to gain significant insights into the cellular effects of [^64^Cu]CuCl_2_ exposure. The study revealed that PCa cells had increased uptake of [^64^Cu]CuCl_2_, which was not due to elevated expression of the main copper importer, hCtr1, as previously suggested. PCa cells also showed higher levels of early DNA damage and genomic instability, with tumor cell lines displaying impaired DNA damage repair following [^64^Cu]CuCl_2_ exposure. This increased cytotoxicity in PCa cells compared to non-cancerous cells supported the notion that [^64^Cu]CuCl_2_ merits further investigation as a theranostic agent for prostate cancer, demonstrating for the first time that PCa cells are more sensitive to [^64^Cu]CuCl_2_ than healthy cells.

Considering that drug evaluation assays conducted in 2D culture systems may yield misleading results because they do not accurately replicate the drug penetration and resistance mechanisms found in in vivo tumors, Pinto et al. (2020) [28] subsequently evaluated the cellular uptake of [^64^Cu]CuCl_2_ in three human PCa spheroids (derived from 22RV1, DU145, and LNCaP cells), and characterized the growth profile and viability of those spheroids as well as the clonogenic capacity of spheroid-derived cells after exposure to [^64^Cu]CuCl_2_. In addition, they assessed in the spheroid models the populations of cancer stem cells (CSCs), which are crucial for cancer resistance and recurrence, using two different markers: CD44 and CD117. This study indicated that [^64^Cu]CuCl_2_ has significant detrimental effects on spheroids and spheroid-derived cells, particularly affecting their growth and impairing their viability and reproductive ability. This effect was observed across both castration-resistant and hormone-naïve prostate cancer (PCa) cell lines. Interestingly, resistance to the treatment with [^64^Cu]CuCl_2_ appears to be correlated with the presence of a cancer stem cell (CSC) population. Spheroids derived from the DU145 cell line, which were the most resistant to [^64^Cu]CuCl_2_ treatment, exhibited the highest initial percentage of CSCs among the three cell lines studied.

In 2023, Serban et al. [29] published their study aimed at investigating the damages caused by [^64^Cu]CuCl_2_ emitted β^−^ particles and Auger electrons in human prostate carcinoma (DU145) cell lines, in addition to colon (HT29 and HCT116) cell lines. They examined the effects on human normal BJ fibroblasts, focusing on the stress responses that underlie cytotoxic and repair mechanisms in the exposed cells. The most striking cytotoxic effects of the radioisotope were observed in colon carcinoma HCT116 cells, showing a substantial decrease in the number of metabolically active cells, along with increased DNA damage and oxidative stress. The in vitro study provides new insights into the molecular mechanisms behind the therapeutic action of [^64^Cu]CuCl_2_ in solid tumors. [^64^Cu]CuCl_2_, with a radioactive concentration of 40 MBq/mL, appears to have therapeutic effects on colon carcinoma, but its use is limited by harmful, yet lesser effects on normal fibroblasts. A lower radioactive concentration of 10–20 MBq/mL might have a partial therapeutic effect on tumor cells, but with reduced radiotoxicity in normal fibroblasts. Colon carcinoma cells attempt to counteract the damaging effects of radiation but can only partially manage the radioactive exposure. Meanwhile, normal human BJ fibroblasts were less reactive or managed to repair the damage within the first 24 h after the addition of [^64^Cu]CuCl_2_. As emphasized by the authors themselves, the primary drawback of in vitro studies lies in the potential inability of cellular models to replicate findings observed in vivo in animal models or cancer patients. Additionally, the presence of Auger–Meitner electrons, which are extremely short-range particles with high linear energy transfer, further restricts the applicability of in vitro findings to complex organisms when the model is fully immersed in an aqueous medium.

### 3.2. Breast Cancer

Kim et al. [30] evaluated the utility of the human copper transporter 1 (*hCTR1*) gene as a new reporter gene for ^64^Cu PET imaging. They infected human breast cancer cells (MDA-MB-231) with a lentiviral vector constitutively expressing the *hCTR1* gene under super cytomegalovirus promoter, and selected positive clones (MDA-MB-231-hCTR1). The expression of the hCTR1 gene in MDA-MB-231-hCTR1 cells was assessed using reverse transcription polymerase chain reaction, Western blot analysis, and a ^64^Cu uptake assay. They also collected small-animal PET images from tumor-bearing mice between 2 and 48 h after administering an intravenous injection of ^64^Cu. The uptake of ^64^Cu in the MDA-MB-231 tumor was 1.368 ± 0.316%ID/g, whereas in the MDA-MB-231-hCTR1 tumor, it was 3.574 ± 0.571%ID/g 48 h after administering ^64^Cu. Over time, the uptake of ^64^Cu in the MDA-MB-231-hCTR1 tumor showed an increasing trend, whereas in the MDA-MB-231 tumor, it remained stable. The liver exhibited the highest uptake of ^64^Cu, as it is primarily involved in copper metabolism; however, this uptake declined sharply over time. Evidence of elevated ^64^Cu uptake resulting from the expression of the hCTR1 gene was shown both in vivo and in vitro. This indicates the potential utilization of the hCTR1 gene as a novel imaging reporter gene for PET imaging with [^64^Cu]CuCl_2_.

### 3.3. Glioblastoma

Glioblastoma multiforme (GBM) is the most prevalent primary malignant brain tumor in adults, with a median survival time of less than one year [31]. To date, there are only a few therapeutic options available for GBM, and their efficacy is limited. The poor penetration of the blood–brain barrier and the high level of GBM chemoresistance result in current chemotherapeutic drugs providing little survival advantage beyond the classical approach of surgery followed by radiotherapy. Due to the increased proliferation rate of glioblastoma, hypoxic regions develop, which are characterized by an elevated concentration of copper (Cu) [32]. In light of this, ^64^Cu has gained attention as a potential theranostic radionuclide for glioblastoma. 

Tumor uptake of ^64^Cu in U87MG (glioblastoma) cell lines in comparison with HT29 (colorectal cancer), FaDu (head and neck cancer) H727 (neuroendocrine lung carcinoid), and A2780 (ovarian cancer) cell lines was studied by Jørgensen et al. [20]. Additionally, they compared copper accumulation in tumor tissue to gene expression of human copper transporter 1 (CTR1). Tumor type-dependent differences of ^64^Cu uptake were observed and three out of five tumor types had a marked increase in uptake between 1 h and 22 h p.i. The tumor uptake of U87MG, HT29, H727, and FaDu xenografts between 2–4%ID/g 1 h p.i and 3–5%ID/g 22 h p.i is at a comparable level to what has previously been reported in xenografts derived from human prostate cancer and extrahepatic hepatomas, having tumor uptake of 3.6 ± 1.3%ID/g and 2.7 ± 0.6%ID/ g 24 h p.i, respectively. However, the tumor uptake of A2780 at 1.3–1.5%ID/g both 1 h and 22 h p.i. is at a significantly lower level. They found no relationship between the tumor uptake of ^64^Cu and gene expression of CTR1.

To provide experimental evidence supporting the application of [^64^Cu]CuCl_2_ to GBM, Ferrari et al. examined the biodistribution, tumor uptake, and therapeutic efficacy of various protocols in xenograft U-87MG cell lines implanted in a mouse model. The study involved injecting animals with [^64^Cu]CuCl_2_ via cardiac injection under anesthesia. The mice were then divided into three groups: a non-treated group, a group treated with a single administration (SDG), and a group treated with a multiple-dose regimen (MDG), where one injection was administered daily for six consecutive days. In nearly all cases, both single- and multiple-dose treatments showed a good response. In the SDG group, the reduction in the volume of interest (VOI) ranged from 68% to 94%, with complete tumor disappearance in two cases. In the MDG group, the VOI reduction ranged from 64% to 92%, with complete tumor disappearance observed in four cases.

Pinto et al. [33] explored the potential of [^64^Cu]CuCl_2_ as a theranostic agent by investigating its effects on advanced three-dimensional models of glioblastoma. The study involved creating spheroids from three types of glioblastoma cells (T98G, U373, and U87), as well as a non-tumoral astrocytic cell line. The therapeutic responses of these spheroids to [^64^Cu]CuCl_2_ exposure were assessed by analyzing their growth, viability, and proliferative capacity. Additionally, the study examined potential mechanisms underlying the therapeutic effects, including the uptake of ^64^Cu, the expression of a copper transporter (CTR1), the presence of cancer stem-like cells, and the production of reactive oxygen species (ROS). Results demonstrated that [^64^Cu]CuCl_2_ significantly reduced the growth and viability of the spheroids, while also impacting their proliferative capacity. The uptake of ^64^Cu, the presence of cancer stem-like cells, and the production of ROS were consistent with the observed therapeutic response. However, the expression levels of CTR1 did not correlate with uptake levels, suggesting the involvement of other mechanisms in ^64^Cu uptake.

### 3.4. Melanoma

Malignant melanoma has become increasingly prevalent and is now a significant health concern in Western countries. This type of cancer is particularly dangerous due to its high propensity to metastasize to various organs, including the lymph nodes, liver, lungs, and brain. Additionally, melanoma exhibits resistance to many common treatment approaches, such as chemotherapy and immunotherapy, contributing to its status as one of the most lethal forms of cancer [34]. Recently, Qin et al. [35] demonstrated the potential of [^64^Cu]CuCl_2_ as a theranostic agent for malignant melanoma. They observed high levels of CTR1 expression in melanoma cells (B16F10, A375M, MDA-MB-435 (26)) and suggested that CTR1 could be a new target for imaging and therapy of malignant melanoma. The treatment with [^64^Cu]CuCl_2_ reduced tumor growth in the studied models compared to the control group. More recently, Jiang et al. [36] presented a pilot study of ^64^Cu(I) for PET imaging of melanoma. The study describes a comparison between ^64^Cu(II) and ^64^Cu(I) in terms of cellular uptake by melanoma. It was found that ^64^Cu(I) had a higher uptake, indicating that CTR1 specifically facilitates the influx of Cu(I). Despite this, due to oxidation reactions occurring in vivo, no significant differences between ^64^Cu(I) and 64Cu(II) were observed in PET images and biodistribution studies.

### 3.5. Wilson’s Disease

Copper deficiency is associated with developmental disorders, anemia and leukopenia, myelopathy, and various neurological disorders [37,38,39,40,41]. However, an excess of copper is harmful to the human body, and the accumulation of copper causes liver tissue damage in Wilson’s disease (WD), a potentially lethal genetic disorder caused by a mutation in the ATP7B gene, which encodes a copper efflux transporter. Although there have been significant advances in understanding the molecular and cellular mechanisms of copper homeostasis, the processes that regulate overall copper metabolism in humans are still not well comprehended [42,43]. 

In 2011, Peng et al. [44] evaluated the gastrointestinal absorption and subsequent biodistribution of copper in knockout mice Atp7b−/−, a well-established mouse model of human WD with PET-CT administered orally as [^64^Cu]CuCl_2_ tracer. As expected, Atp7b−/− knockout mice exhibited increased hepatic uptake of ^64^Cu following the oral administration of [^64^Cu]CuCl_2_. Twenty-four hours after administration, a high level of ^64^Cu radioactivity was observed in the liver of Atp7b−/− knockout mice, whereas no such radioactivity was detected in the liver of control C57BL WT mice.

In a follow-up study [45], the same researchers explored the use of [^64^Cu]CuCl_2_ as a tracer to noninvasively monitor age-related alterations in brain copper metabolism associated with Wilson’s disease. They employed an Atp7b−/− knockout mouse model and used positron emission tomography/computed tomography (PET/CT) for imaging. Their quantitative PET analysis showed that at 7 weeks old, the brains of Atp7b−/− knockout mice had low levels of ^64^Cu radioactivity compared to the brains of C57BL/6 wild-type mice, measured 24 h after an intravenous injection of [^64^Cu]CuCl_2_. Additionally, an age-related increase in ^64^Cu radioactivity was observed in the brains of Atp7b−/− knockout mice between 13 and 21 weeks of age. These findings indicate that [^64^Cu]CuCl_2_ PET/CT imaging can be clinically useful for non-invasive evaluation of age-related changes in brain copper metabolism in Wilson’s disease patients, who exhibit a range of neurological and psychiatric symptoms. 

### 3.6. Lung Cancer

Lung cancer is the most common and deadliest cancer globally, responsible for 23.5% of cancer deaths in 2019. Despite advancements in treatments such as thoracoscopic surgery, chemotherapy, radiotherapy, targeted therapy, and immunotherapy, the overall survival rate remains 5 years [46]. Hence, an efficient non-invasive technique for the accurate diagnosis of lung cancer is important to help clinicians choose the proper treatment and prolong patients’ lives.

Wang et al. [47] identified the expression of the human copper transporter 1 (CTR1) in a series of lung cancer cell lines using a quantitative real-time polymerase chain reaction (Q-PCR), Western blot, enzyme-linked immunosorbent assay (ELISA), and immunofluorescent staining. The results showed that the H1299 cell line had high CTR1 expression, while the H460 and H1299 cell lines expressed low to moderate levels of CTR1. Therefore, a positive correlation between ^64^CuCl_2_ uptake and CTR1 expression levels was shown in the H1299, H460, and H1703 cell lines, which were selected for their high, moderate, and low CTR1 expression, respectively. Blocking experiments confirmed the specificity of ^64^CuCl_2_ in targeting CTR1. In the same study [47], small animal PET imaging on tumor-bearing mice of H1299, H460, and H1703 demonstrated that ^64^CuCl_2_ accumulation matched the CTR1 levels and in vitro cellular uptake findings. Small animal PET data of H1299 tumor-bearing mice has shown that ^64^CuCl_2_ had rapid and high tumor uptake, rapid clearance from blood and most normal tissues, and high tumor-to-normal tissue ratios, suggesting the potential of [^64^Cu]CuCl_2_ as a promising PET imaging agent of lung cancer. In 2020, García–Pérez et al. [48] developed appropriate clinical protocols for imaging or assessing patients with lung cancer and Parkinson’s disease using the theranostic agent [^64^Cu]CuCl_2_. The results of their study suggest that [^64^Cu]CuCl_2_ PET/CT has a good ability to detect lesions, as the detection rate of [^64^Cu]CuCl_2_ PET/CT was not statistically different from that of [^18^F]FDG PET/CT. Additionally, [^64^Cu]CuCl_2_ uptake is based on the expression of Ctr1 transporters, which may help differentiate patients who could benefit from platinum-based therapy.

## 4. Conclusions

The studies reported in this review suggest the potential of the [^64^Cu]Cu^2+^ ion for theranostic applications, where a single compound can serve both diagnostic and therapeutic purposes. These highlight the importance of further exploring the potential of [^64^Cu]CuCl_2_ in the context of prostate cancer, glioblastoma treatment, and diagnosis as well as other solid tumors.

Further studies are needed to understand the molecular mechanisms by which ^64^Cu acts differentially on tumor and normal cells, as well as the induced stress responses. This understanding can aid in designing an enhanced theragnostic strategy for cancer treatment.

## Figures and Tables

**Table 1:** Nuclear proprieties of the most representative copper radionuclides.

Radionuclide	T_1/2_	E_βmean_ (keV, int.%)	Eγ (keV, int.%)	E _Auger_ (keV, int.%)
^61^Cu	3.33 h	500 (61)	283 (12)	0.84 (52)
			511 (123)	6.54 (20)
			656 (11)	
			1185 (4)	
^64^Cu	12.7 h	β^+^, 278 (17.6) β^−^, 19 (38.5)	511 (32)	0.84 (58)
			1346 (0.5)	6.54 (22)
^67^Cu	61.8 h	β^−^, 141 (11)	91 (7)	0.99 (19)
			93 (16)	7.53 (7)
			185 (49)	83.65 (12)

## Data Availability

Not applicable.
